# Adolescents with close relatives affected by severe health conditions: prevalence and psychosocial correlates of mental well-being in a Swedish cross-sectional school-based study

**DOI:** 10.1136/bmjpo-2026-004632

**Published:** 2026-08-13

**Authors:** Eva Van Geerenstein, Veronica Hermann, Fredrik Söderqvist

**Affiliations:** 1 Hällefors Primary Health Care Centre, Region Örebro län, Örebro, Sweden; 2 Child Health And Parenting (CHAP), Uppsala University, Uppsala, Sweden; 3 University Health Care Research Centre, Faculty of Medicine and Health, Örebro universitet, Örebro, Sweden

**Keywords:** Adolescent Health, Cross-Sectional Studies, Epidemiology

## Abstract

**Background:**

Adolescents living with a close relative with severe health conditions (RSHC) may experience challenges affecting their mental well-being. Previous research has largely focused on caregiving roles or specific illnesses while less is known about broader populations of adolescents with RSHC. This study aimed to describe the prevalence of adolescents with RSHC and to examine associations with mental well-being across different sociodemographic and psychosocial contexts.

**Methods:**

The data were drawn from a cross-sectional, population-based school survey conducted in Örebro County, Sweden, which included 4 140 adolescents aged 15–18 years. Mental well-being was assessed using the Mental Health Continuum–Short Form. Sequentially adjusted linear regression stratified by gender was used to examine associations between RSHC and mental well-being, accounting for key factors.

**Results:**

Half of the adolescents (n=2 212) reported having at least one RSHC, with 31% reporting one and 19% reporting several RSHCs. Adolescents with RSHC reported significantly lower mean mental well-being than those without RSHC. In the fully adjusted model, substance abuse in a close relative remained associated with lower mental well-being, whereas associations for other RSHC types were attenuated. General stress and perceived availability of someone to confide in were strongly associated with mental well-being.

**Conclusion:**

Having a relative with RSHC is common among adolescents and is associated with lower mental well-being, particularly when close relatives are affected by substance abuse. The findings suggest that adolescent mental well-being in the context of RSHC is shaped by broader psychosocial factors, including stress and available support.

**WHAT IS ALREADY KNOWN ON THIS TOPIC**

- Previous research has mainly focused on young carers or on adolescents with parents affected by specific illnesses, often based on clinical or hospital-based samples.

**WHAT THIS STUDY ADDS**

- This population-based study shows that living with a close relative with severe health conditions is common among adolescents and associated with lower mental well-being, particularly when close relatives are affected by substance use.

**HOW THIS STUDY MIGHT AFFECT RESEARCH, PRACTICE OR POLICY**

- The findings highlight adolescents with RSHC as a broad and heterogeneous group, extending beyond those identified as young carers, and underline the importance of recognising psychosocial stress and access to available support when identifying adolescents at risk.

## Introduction

 Many children and adolescents grow up with close relatives who have chronic physical or mental illnesses, disabilities or substance use problems. In a recent population-based study conducted in Sörmland County, Sweden, more than half of the adolescents reported having at least one close relative with a severe health condition (RSHC).^1^ This finding underscores the widespread nature of this issue and its potential significance for adolescent development.

Adolescents with RSHC may take on caregiving responsibilities and thereby become young carers, although not all adolescents with RSHC engage in such caregiving. While caregiving is an important aspect of this context, the present study focuses more broadly on adolescents with RSHC regardless of caregiving involvement.^2^ When present, caregiving may involve responsibilities that are inappropriate for the adolescent’s age and developmental stage, potentially affecting mental health and well-being. International estimates suggest that 2–8% of children and adolescents are young carers, with somewhat higher proportions reported in Swedish regional data.^3–5^ Caregiving responsibilities have been associated with both positive outcomes, such as increased independence and empathy, and negative outcomes, including social isolation, reduced participation in school and peer activities and increased stress.^3–7^

However, adolescents with RSHC who do not take on caregiving responsibilities may still be affected by the presence of illness in close relations. Consequently, adolescents with RSHC represent a vulnerable group that may experience social, emotional and educational disadvantages compared with their peers, regardless of caregiving involvement, with potential implications for their mental health.

Mental health is increasingly conceptualised not merely as the absence of psychological difficulties or psychiatric illness but also as the presence of positive dimensions of well-being. This view aligns with the WHO’s definition of health as ‘a state of complete physical, mental, and social well-being and not merely the absence of disease or infirmity’.^8^ The WHO further defines mental health as ‘a state of mental well-being that enables people to cope with the stresses of life, realize their abilities, learn well and work well, and contribute to their community’^9^ underscoring its multidimensional character and its dynamic interaction with the surrounding environment. An operationalisation of mental well-being that corresponds closely to this definition has been proposed by Keyes, encompassing three domains of well-being: the presence of positive feelings (emotional well-being) and positive functioning in individual life (psychological well-being) and community life (social well-being).^10^

Mental well-being is shaped by multiple sociodemographic and contextual factors. Previous research has shown that boys generally report higher levels of mental well-being than girls.^11^ Ethnicity, family socioeconomic conditions and school achievement have also been associated with mental well-being.^11–14^ Against this background, the overall objective of this study was to examine the mental well-being among adolescents with RSHC in the county of Örebro, Sweden. Specifically, the study aimed (1) to describe the prevalence of adolescents with RSHC in the county; (2) to examine differences in mental well-being across groups defined by key background factors; (3) to explore the association between RSHC and mental well-being and (4) to examine whether adolescent characteristics—such as gender, grade, economic stress and ethnicity—account for or attenuate this association.

Based on previous research, it was hypothesised that adolescents with RSHC would report lower levels of mental well-being than their peers, and that this association would remain significant after adjustment for relevant covariates.

## Methods

### Study design

The data were extracted from the Life and Health Youth Survey, a cross-sectional school-based study performed tri-annually in the county of Örebro, Sweden. The objective of this population-based survey is to monitor public health in young people. The survey was designed as a total population study and was distributed to all students attending the ninth grade in ordinary lower secondary school and the second year of upper secondary school programmes. Students enrolled in introduction programmes or special education schools were not included. The questionnaire included approximately 88 questions.

### Setting and population

Data collection took place between January and March 2023. Students completed a web-based questionnaire anonymously during school hours in a supervised classroom setting. Participation was voluntary and contingent on students being present in school on the day of the survey and completing the questionnaire.

The 2023 questionnaire was answered by 4457 students, a response rate of 68%. Of these students, 2500 were in the ninth grade (response rate 71%) and 1957 in the second year of upper secondary school (response rate 65%). The question about having close relatives with severe health conditions was answered by 4386 students. Of these students, 246 were excluded from analyses involving mental well-being due to incomplete data regarding mental well-being as measured by the Mental Health Continuum Short Form (MHC-SF). Outcome and item-level missing data ranged from 0.9% to 2.4% for explanatory variables and up to 6.5% for the outcome; analyses were therefore conducted using complete-case data.

### Patient and public involvement

Patients and/or the public were not involved in the design, conduct, reporting or dissemination plans of this research.

### Measures and variables

The questionnaire had the following question related to RSHC: do you have a family member or a person close to you that has

1. a severe physical illness/injury/impairment;
2. a severe mental illness and/or a psychiatric disorder/impairment;
3. a substance abuse or gambling;
4. died.

The question did not specify the role of the relative (eg, primary caregiver) and may therefore include a range of close relationships.

Mental well-being was measured using Keyes’ MHC-SF, a validated 14-item tool capturing three domains of well-being: emotional, psychological and social well-being.^10 14–16^ Items are rated based on the frequency of symptoms experienced during the past month, on a six-point scale from 0 (never) to 5 (every day), with a total score ranging from 0 to 70. A higher score is equivalent to a higher level of well-being.^11^ The MHC-SF scores can be used as a continuous measure for well-being or to categorise mental well-being into two (flourishing and languishing) or three (flourishing, moderate and languishing) different states. In the present study, mean MHC-SF scores were analysed as a continuous outcome.

A number of items from the questionnaire were used in order to measure background factors associated with mental health and/or mental well-being. These covariates included: gender, ethnicity, economic stress, given the grade F or no grade in any subjects, feeling stressed, neurodevelopmental or physical disability and perceived availability of someone to confide in. Economic stress is a combination of two questions: (1) do you worry about your family’s economy and (2) can you afford the same expenses as others your age. An overview of the questions used in the present study is presented in online supplemental appendix A. The covariates were selected to capture key sociodemographic and contextual factors relevant to inequalities in adolescent mental well-being, in line with previous population-based research on the distribution of well-being across social groups.

### Statistical analysis

Frequencies and percentages were used to describe the prevalence of RSHC. Pearson’s χ^2^ test was used to determine differences in background factors between the group with and without RSHC.

Means and SD were used to describe the MHC-SF scores in the group with and without RSHC and in different groups based on gender, grade, ethnicity, economic stress and physical and neurodevelopmental disability. The corresponding differences in well-being were analysed using independent samples t-tests for binary variables and Welch’s analysis of variance for variables with more than two categories. Effect sizes for significant differences in MHC-SF were expressed as Cohen’s d for binary variables and eta squared (η²) for variables with more than two categories.

Sequentially adjusted linear regression models were used to examine the relationship between having RSHC experiences and adolescents’ mental well-being. Three models were estimated. Model 1 examined the crude association between the four types of RSHC exposure. Model 2 added sociodemographic and individual-level characteristics (ethnicity, grade, physical disability, neurodevelopmental disability and economic stress) to assess whether associations persisted after adjustment for these factors. Model 3 further included psychosocial factors—general stress, school performance and emotional support—to explore whether these factors accounted for additional variance in mental well-being.

Given previous research^11 12^ indicating gender and stress differences in adolescent well-being, regression models were stratified by gender to explore potential variations in the strength of associations. Inspection of P-P and scatter plots of standardised residuals indicated approximate normality and homoscedasticity across all models and strata; model assumptions were therefore considered adequately met. Multicollinearity was assessed using variance inflation factors (VIFs), with no evidence of problematic multicollinearity (all VIF values <1.3).

Model p values, R², ΔR² and changes in R^2^ are reported together with unstandardised regression (B) coefficients to illustrate the main effect of the covariates. All statistical analyses were conducted using SPSS (V.29).

### Reporting guideline

We used the Strengthening the Reporting of Observational Studies in Epidemiology (STROBE) reporting guideline^17^ to draft this manuscript and the STROBE reporting checklist^18^ when editing.

## Results

### The prevalence of having RSHC and background characteristics of adolescents

This study included 4 386 adolescents, 2 454 in the ninth grade and 1 932 in the second year of upper secondary school. Half of the adolescents (n=2 212) reported having at least one RSHC, with 31% reporting one RSHC and 19% reporting several RSHCs. Of the adolescents having one or several RSHC, 45% reported having a close relative who had died, representing the largest group. This was followed by adolescents reporting a close relative with a mental illness or psychiatric disorder (24%), a physical illness or impairment (18%) and a substance abuse or gambling (13%) (table 1, panel A). The prevalence of having close RSHCs and the distribution of the different types of RSHCs were similar among adolescents in the ninth grade (year 9) and those in the second year of upper secondary school (year 2 upper secondary (US)).

**Table 1:** Prevalence of RSHC and background characteristics of adolescents

Panel A: prevalence of RSHC and subtype distribution			
RSHC exposure	Total N (%)	Year 9, n (%)	Year 2 US, N (%)
No RSHC	2174 (50%)	1202 (49%)	972 (50%)
One RSHC	1370 (31%)	766 (31%)	604 (31%)
Several RSHC	842 (19%)	486 (20%)	356 (19%)
RSHC type among those with ≥1 RSHC			
Physical illness	618 (18%)	372 (19%)	246 (17%)
Mental illness	843 (24%)	461 (23%)	382 (26%)
Substance abuse or gambling	445 (13%)	262 (13%)	183 (12%)
Death	1561 (45%)	898 (45%)	663 (45%)

Panel B: background characteristics by RSHC status					
Variable	Category	Total n	No RSHC	≥1 RSHC	P value
			n (%)	n (%)	
Gender	Boy	2064	1169 (54%)	895 (42%)	<0.001
	Girl	2200	976 (46%)	1233 (58%)	
Grade	Year 9	2454	1202 (55%)	1252 (57%)	0.4
	Year 2 US	1932	972 (45%)	960 (43%)	
Ethnicity	Swedish	3640	1737 (81%)	1903 (87%)	<0.001
	Other	684	409 (19%)	275 (13%)	
Economic stress	No	3676	1930 (89%)	1746 (79%)	<0.001
	Yes	703	240 (11%)	463 (21%)	
Physical disability	No	3848	1953 (90%)	1895 (86%)	<0.001
	Yes	512	208 (10%)	304 (14%)	
Neurodevelopmental disability	No	3359	1787 (83%)	1572 (72%)	<0.001
	Yes	995	371 (17%)	624 (28%)	
Stress	No	2550	1453 (67%)	1097 (50%)	<0.001
	Yes	1824	714 (33%)	1110 (50%)	
Failing grades	No	3164	1620 (76%)	1544 (71%)	<0.001
	Yes	1142	514 (24%)	628 (29%)	
Someone to confide in	No	540	229 (11%)	311 (14%)	<0.001
	Unsure	877	377 (17%)	500 (23%)	
	Yes	2959	1564 (72%)	1395 (63%)	

Values represent observed proportions within each group. Group differences were assessed using Pearson’s χ2 test.

RSHC, relative with a severe health condition; US, upper secondary school.

Descriptive statistics for adolescents with and without RSHC are reported in table 1, panel B. Girls accounted for 58% of adolescents with at least one RSHC. Compared with adolescents without RSHC, a higher proportion of those with at least one RSHC were of Swedish ethnicity (87% vs 81%). Adolescents who reported having at least one RSHC more frequently reported economic stress, failing grades and general stress compared with adolescents without RSHC. In addition, adolescents with RSHC more often reported having a physical or neurodevelopmental disability and less often reported having someone to confide in.

### Adolescents with RSHC and their mental well-being

The questions about having a close RSHC and mental well-being were answered by 4 140 adolescents. Table 2 presents mean scores and SD for mental well-being as measured by the MHC-SF across key subgroups. Mean scores for adolescents with at least one RSHC were significantly lower than among adolescents without RSHC: 39.46 (SD 15.10) compared with 44.01 (SD 14.76) among adolescents without RSHC. Mental well-being also differed significantly by gender. Boys reported higher mean scores than girls (44.66 vs 39.57, respectively).

**Table 2:** Mean mental well-being (MHC-SF) across groups of adolescents

Variable	Group	N	(M±SD)	P value	Effect size
RSHC				<0.001	0.31
	No	2044	44.01 (14.76)		
	Yes	2096	39.46 (15.10)		
Gender				<0.001	0.35
	Boy	1966	44.66 (15.08)		
	Girl	2080	39.57 (14.10)		
Grade				0.46	–
	Year 9	2296	41.52 (15.56)		
	Year 2 US	1870	41.86 (14.60)		
Ethnicity				0.10	–
	Swedish	3486	41.69 (14.76)		
	Other	624	42.82 (15.89)		
Economic stress				<0.001	0.60
	No	3499	42.80 (14.82)		
	Yes	524	33.87 (15.05)		
Physical disability				<0.001	0.32
	No	3678	42.24 (14.74)		
	Yes	478	37.42 (17.22)		
Neurodevelopmental disability				<0.001	0.31
	No	3226	42.69 (14.59)		
	Yes	922	38.12 (16.39)		
General stress				<0.001	0.61
	Low	1195	48.04 (15.10)		
	High	2965	39.14 (14.35)		
Failing grades				<0.001	0.28
	No	3047	42.77 (14.41)		
	Yes	1057	38.63 (16.56)		
Someone to confide in				<0.001	0.13
	No	513	31.91 (15.37)		
	Unsure	815	34.98 (14.13)		
	Yes	2834	45.39 (13.84)		

Values represent mean scores (M±SD) on the MHC-SF. Group differences were tested using independent sample t-tests for binary variables and Welch’s analysis of variance for variables with more than two categories. Effect sizes are reported as Cohen’s d (0.2 = small, 0.5 = medium, 0.8 = large) or eta squared (η²; 0.01 = small, 0.06 = medium, 0.14 = large).

MHC-SF, Mental Health Continuum-Short Form; RSHC, relative with a severe health condition; US, upper secondary school.

Most of the background factors that were more prevalent among adolescents with at least one RSHC were also associated with lower mean MHC-SF scores, including economic stress, physical disability, neurodevelopmental disability, general stress and failing grades. No statistically significant differences in mean mental well-being were observed across groups defined by ethnicity or school grade. Perceived availability of someone to confide in was strongly associated with mental well-being. Mean MHC-SF scores increased stepwise across categories, with the lowest scores observed among adolescents reporting no one to confide in, intermediate scores among those who were unsure and the highest scores among those reporting having someone to confide in.

### Association between RSHC and adolescents’ mental well-being

The sequentially adjusted linear regression models examining the associations between RSHC and adolescents’ mental well-being, stratified by gender, are presented in table 3. In model 1, several RSHC-types were associated with lower levels of mental well-being. Among boys, negative associations were observed for having a close relative with a physical illness, mental illness or substance abuse, whereas among girls, significant associations were observed for mental

**Table 3:** Sequentially adjusted linear regression models of the association between RSHC and adolescents’ mental well−being stratified by gender

Predictors	Model 1, B (95% CI)		Model 2, B (95% CI)		Model 3, B (95% CI)	
	Boys	Girls	Boys	Girls	Boys	Girls
RSHC: physical illness	−2.516 (−4.801 to −0.232)	−0.596 (−2.293 to 1.100)	−1.536 (−3.802 to 0.730)	−0.328 (−1.993 to 1.336)	−1.156 (−3.261 to 0.949)	−0.676 (−2.212 to 0.860)
RSHC: mental illness	−3.368 (−5.480 to −1.256)	−3.516 (−5.000 to −2.032)	−2.377 (−4.474 to −0.280)	−2.480 (−3.957 to −1.004)	−1.497 (−3.448 to 0.454)	−0.827 (−2.202 to 0.548)
RSHC: substance abuse or gambling	−5.487 (−8.274 to −2.699)	−5.039 (−6.969 to −3.109)	−4.654 (−7.413 to −1.895)	−4.023 (−5.926 to −2.121)	−2.909 (−5.480 to −0.339)	−2.543 (−4.310 to −0.777)
RSHC: death	0.168 (−1.280 to 1.616)	−2.127 (−3.391 to −0.863)	0.590 (−0.842 to 2.022)	−1.842 (−3.082 to −0.603)	1.035 (−0.296 to 2.365)	−1.415 (−2.561 to −0.270)
Ethnicity			2.031 (0.172 to 3.890)	1.228 (−0.461 to 2.918)	2.747 (0.993 to 4.502)	0.579 (−1.003 to 2.160)
Grade			−1.213 (−2.526 to 0.101)	1.575 (0.398 to 2.752)	−1.237 (−2.463 to −0.012)	1.524 (0.429 to 2.620)
Physical disability			−1.481 (−3.653 to 0.691)	−1.854 (−3.799 to 0.092)	−1.086 (−3.102 to 0.930)	−1.121 (−2.919 to 0.677)
Neurodevelopmental disability			−3.271 (−4.880 to −1.662)	−1.422 (−2.944 to 0.099)	−2.161 (−3.682 to −0.640)	−1.232 (−2.672 to 0.207)
Economic stress			−5.931 (−8.030 to −3.832)	−7.000 (−8.584 to −5.415)	−2.771 (−4.753 to −0.790)	−4.838 (−6.317 to −3.359)
Someone to confide in					6.378 (5.462 to 7.293)	5.329 (4.541 to 6.117)
Failing grades					−2.714 (−4.150 to −1.278)	−2.788 (−4.136 to −1.439)
General stress					−6.905 (−8.448 to −5.362)	−6.382 (−7.526 to −5.237)
R²	0.024	0.044	0.055	0.088	0.188	0.225
ΔR²	0.024	0.044	0.031	0.044	0.133	0.137
Model p value	<0.001	<0.001	<0.001	<0.001	<0.001	<0.001

Values are unstandardised regression coefficients (B) with 95% CIs. Negative B values indicate lower mental well-being as measured by the MHC-SF. Model 1 includes RSHC types only. Model 2 is additionally adjusted for sociodemographic and individual-level factors (ethnicity, grade, physical disability, neurodevelopmental disability and economic stress). Model 3 is further adjusted for psychosocial factors (general stress, failing grades and perceived availability of someone to confide in). Models were stratified by gender. ΔR² indicates the change in explained variance compared with the previous model.

MHC-SF, Mental Health Continuum-Short Form; RSHC, relative with a severe health condition.

illness, substance abuse and death of a close relative.

In model 2, which adjusted for sociodemographic characteristics, the pattern of association remained largely unchanged among girls, while among boys, only mental illness and substance abuse remained significantly associated with lower mental well-being.

**Figure 1:**
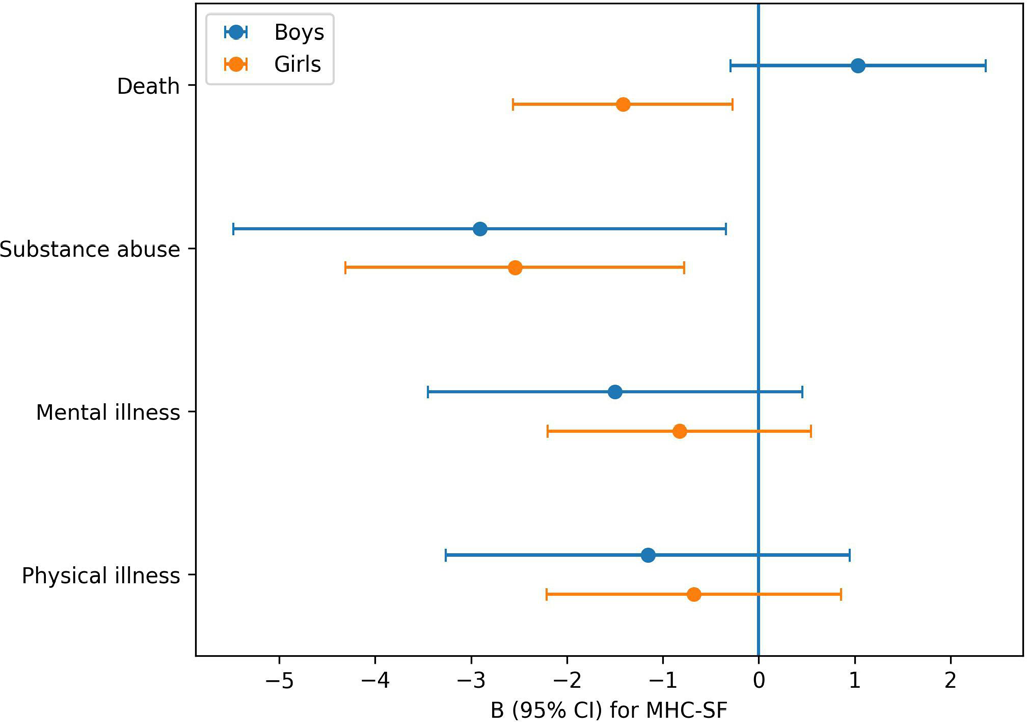
Adjusted associations between different types of relatives with severe health conditions and adolescent mental well-being. Points represent unstandardised regression coefficients from the fully adjusted model (model 3), stratified by gender, with 95% CIs. Negative values indicate lower mental well-being (MHC-SF score). MHC-SF, Mental Health Continuum-Short Form.

In the fully adjusted model 3, having a close relative with substance abuse remained significantly associated with lower mental well-being in both boys and girls, representing the most consistent RSHC-related association across models (boys: B −2.909, 95% CI −5.480 to −0.339; girls: B −2.543, 95% CI −4.310 to −0.777). Having a close relative who had died remained significantly associated with lower mental well-being only among girls. The adjusted associations between different RSHC types and mental well-being are illustrated in figure 1. Overall, associations with mental well-being differed across RSHC subtypes, with having a close relative with substance abuse showing the strongest association with lower mental well-being, whereas having a close relative who had died showed the weakest association.

Several background and psychosocial factors showed strong associations with mental well-being in model 3. General stress was strongly and negatively associated with mental well-being in both boys and girls, with larger effect estimates than any individual RSHC exposure. In contrast, perceived availability of someone to confide in was positively associated with mental well-being in both genders.

School grade showed opposite associations by gender, with a small negative association among boys and a positive association among girls. Economic stress and failing grades were negatively associated with mental well-being in both boys and girls. Ethnicity and neurodevelopmental disability showed significant associations with mental well-being only among boys.

The explanatory power of the models increased substantially with sequential adjustment. In boys, R² increased from 0.024 in Model 1 to 0.188 in model 3, and in girls from 0.044 to 0.225, with the largest increase in explained variance observed after inclusion of psychosocial factors (ΔR² = 0.133 in boys and 0.137 in girls). All models were statistically significant (model p value <0.001).

## Discussion

The present study aimed to describe the prevalence of adolescents with RSHC and to examine how such experiences are associated with adolescent mental well-being across different groups. In line with these aims, the findings indicate that RSHC is common among adolescents and that mental well-being differs across several background characteristics, both in relation to and beyond RSHC exposure itself.

Consistent with previous Swedish studies, approximately half of the adolescents reported having at least one close RSHC, underscoring the widespread nature of these experiences.^1 19^ However, it is important to note that having a close relative with RSHC does not necessarily imply being a young carer, as not all adolescents with RSHC undertake caregiving responsibilities. Since this study did not include specific questions on caregiving activities, it is not possible to further explore the overlap between RSHC exposure and young caregiving, which remains an important area for future research.

Beyond prevalence, adolescents with RSHC were more likely to be situated in a vulnerable context, both at home and at school. Adolescents with RSHC more frequently reported economic stress and general stress and were also more likely to report failing grades. These findings align with previous research indicating that families affected by chronic illness often experience financial strain and broader psychosocial challenges.^3 7 19–21^ Taken together, these results support a broader perspective on mental well-being as socially patterned, where differences between groups reflect underlying inequalities in living conditions and access to resources.^13^ Such contextual factors are likely to shape adolescents’ everyday functioning and may contribute to differences in mental well-being observed between adolescents with and without RSHC. For example, previous research has shown that almost every second family including a parent with a chronic medical condition received less income after the diagnosis.^7^ Low income has been identified as a factor influencing both the likelihood of becoming a young carer and the level of caregiving undertaken.^3^

Importantly, this study also highlights that RSHC should be understood as a heterogeneous exposure rather than a uniform experience. While previous research has often focused on specific diagnoses—most commonly cancer—this study included a broader range of severe health conditions affecting close relatives.^7^ The findings suggest that different types of RSHC are associated with mental well-being to varying degrees, with having a close relative with substance abuse showing the strongest association with lower well-being, whereas having a close relative who had died showed a comparatively smaller association.

When psychosocial factors were included in the fully adjusted model, the associations between several RSHC types and mental well-being were attenuated rather than eliminated. This pattern suggests a shift in explanatory emphasis, where the presence of RSHC alone accounted for less of the variation in mental well-being once broader contextual factors were considered. In these models, general stress emerged as the strongest factor associated with lower mental well-being, with effect estimates exceeding those observed for any individual RSHC exposure and showing similar magnitude in both boys and girls.

At the same time, perceived availability of someone to confide in was consistently associated with higher levels of mental well-being across models and genders, indicating a potentially important protective resource. In this context, these findings suggest that adolescents with RSHC experience lower mental well-being not solely due to the presence of RSHC, but within a broader psychosocial context characterised by elevated stress and varying access to emotional support. From this perspective, the observed attenuation of RSHC-related associations suggests that RSHC interacts with everyday stressors and available resources in shaping adolescents’ mental well-being, rather than functioning as an isolated determinant.

From a practical perspective, these findings point to the importance of supportive resources in adolescents’ everyday environments. Existing interventions have primarily focused on specific subgroups, such as young carers or children of parents with mental illness or substance use problems, including psychosocial, family-based and school-linked support programmes.^22–24^ However, fewer interventions appear to target adolescents with RSHC as a broader and heterogeneous group, highlighting a need for support strategies that reflect the diversity of these experiences and the importance of emotional support and everyday psychosocial context.

Given well-documented gender differences in adolescent mental well-being and stress exposure, analyses were stratified by gender to allow associations to be examined separately for boys and girls; a pooled model adjusting for gender would have provided an average estimate across genders and would require interaction terms to formally assess effect modification.^12 14 15^ Overall, the stratified analyses revealed largely similar patterns of association between RSHC and mental well-being in boys and girls. In both genders, general stress and perceived availability of someone to confide in showed strong associations with mental well-being, and the inclusion of psychosocial factors led to attenuation of several RSHC-related associations.

Some differences in magnitude were nonetheless observed. For example, having a close relative who had died was associated with lower mental well-being among girls but not among boys, whereas other RSHC types showed broadly comparable patterns across genders. Taken together, these findings suggest that gender differences primarily reflect variations in the strength of associations rather than fundamentally different patterns, indicating that the psychosocial context surrounding RSHC exposure may operate in similar ways for boys and girls.

### Strengths and limitations

A strength of the study was its large sample size of 4 386 adolescents and its population-based study design, which supports generalisability to Swedish adolescents and, with some caution, to other similar populations. A further strength of this study is the use of the validated MHC-SF, which has demonstrated good psychometric properties in previous research.^25 26^ In the present study, mean MHC-SF scores were used as a continuous outcome, where higher scores correspond to higher levels of mental well-being.

Several limitations should also be considered. One of the limitations of this study was the measurement of having a close RSHC. The question did not specify the type of relationship between the close relative and the adolescent. The interpretation of what constitutes a close relative was therefore subjective and may have led to the inclusion of a heterogeneous range of relationships. At the same time, if an adolescent considers a person to be a close relative, this likely reflects emotional closeness, which may be relevant for understanding potential impacts on their everyday life. In addition, the question may have been perceived as sensitive and could have contributed to non-response and selection bias among adolescents who otherwise would have been eligible for inclusion. Missing data on other covariates may also have led to an underestimation of associations observed in the present study. Although the MHC-SF is a validated instrument, mental well-being was assessed using self-report only, and no complementary clinical assessments were available for triangulation.

Finally, as in all cross-sectional studies, the results in the current study reflect associations rather than causal relationships between RSHC and mental well-being.

## Conclusions

This study indicates that adolescents with RSHC represent a vulnerable group with lower levels of mental well-being compared with their peers. Associations were particularly pronounced among adolescents with RSHC related to mental illness or substance abuse. These findings highlight the importance of recognising adolescents with RSHC in clinical and educational settings. Early identification of this group may help inform timely and appropriate support, with potential relevance for adolescents’ well-being and developmental opportunities.

## Supplementary material

10.1136/bmjpo-2026-004632 online supplemental file 1

## Data Availability

Data are available upon reasonable request.
