# Supplementary material for: Adolescents with close relatives affected by severe health conditions: prevalence and psychosocial correlates of mental well-being in a Swedish cross-sectional school-based study
Source: BMJ Paediatr Open. 2026 Aug 13;10(1):e004632. doi: 10.1136/bmjpo-2026-004632 (PMC13475572; doi:10.1136/bmjpo-2026-004632)
Supplement: Supplementary file 1 [file bmjpo-10-1-s001.pdf]

Appendix A **Life and Health Youth questions used in this study**

Questions in the survey	Independent variables in the analysis
What is your gender?	**Gender**<br>Girl<br>Boy
Where are you born?	**Ethnicity**<br>Sweden<br>Other (Northern Europe, Other countries)
Do you have someone you can talk to about your innermost feelings?	**Someone to confide in**<br>No<br>Unsure<br>Yes
Do you have a family member or a person close to you that has	
a severe physical illness/injury/impairment	**RSHC: Physical illness**<br>No<br>Yes
b A severe mental illness and/or a psychiatric disorder/impairment	**RSHC: Mental illness**<br>No<br>Yes
c A substance abuse or gambling disorder	**RSHC: Substance abuse**<br>No<br>Yes
d Died	**RSHC:Death**<br>No<br>Yes
Are you worried for your family's economy	**Economic stress**<br>No (Not worried at all, Not especially worried)<br>Yes (Yes, quite; Yes, very worried)
During the past three months, how often have you been feeling stressed?	**General stress**<br>No (Never, Seldom, Sometimes)<br>Yes (Often, Every day)
Do you have a failing grade, or do you lack grades in any subject?	**Failing grades**<br>No<br>Yes (Yes, in 1-2 subjects;Yes, in 3-4 subjects; Yes, in 5 or more subjects
Do you have one of the following disabilities?<br>a Hearing impairment, b Visual impairment, c Mobility disability, d Learning disability (dyslexia, dyscalculia), e ADHD or ADD, f Autism or autism spectrum disorder (ASD)	**Physical disability**<br>No<br>Yes (on any of a–c)<br><br>**Neurodevelopmental disability**<br>No<br>Yes (on any of d–f)
