# Supplementary material for: Adolescents with close relatives affected by severe health conditions: prevalence and psychosocial correlates of mental well-being in a Swedish cross-sectional school-based study
Source: BMJ Paediatr Open. 2026 Aug 13;10(1):e004632. doi: 10.1136/bmjpo-2026-004632 (PMC13475572; doi:10.1136/bmjpo-2026-004632)
Supplement: Supplementary file 2 [file bmjpo-2026-004632.reviewer_comments.pdf]

PEER REVIEW HISTORY

BMJ Paediatrics Open publishes all reviews undertaken for accepted manuscripts. Reviewers are asked to complete a checklist review form and are provided with free text boxes to elaborate on their assessment. These free text comments are reproduced below.

ARTICLE DETAILS

**TITLE (PROVISIONAL)**	Adolescents with close relatives affected by severe health conditions: Prevalence and psychosocial correlates of mental well-being in a Swedish cross-sectional school-based study
**AUTHORS**	Van Geerenstein, Eva<br>Hermann, Veronica<br>Söderqvist, Fredrik

VERSION 1 – REVIEW

**REVIEWER NAME**	Thazhissery, Anjana
**REVIEWER AFFILIATION**	Sydney South West Area Health Service, ICAMHS
**REVIEWER CONFLICT OF INTEREST**	No Conflict of Interest
**AI DISCLOSURE**	No, I have not used AI
**DATE REVIEW RETURNED**	11-Mar-2026

**GENERAL COMMENTS**	Thank you for providing me with the oppurtunity to review your study. This is a very interesting relevant study with focus on the social determinants of adolescent mental health. I do have a couple of queries:<br>1) is the "close relative" identified as the primary carer ofr the adolescent.<br>2) given the response rate, is the catchment area, socio-economically disadvantaged ? is this a culturally and linguistically diverse cohot?

**REVIEWER NAME**	Tiikkaja, Sanna
**REVIEWER AFFILIATION**	Uppsala University Department of Public Health and Caring Sciences, CHAP
**REVIEWER CONFLICT OF INTEREST**	No Conflict of Interest
**AI DISCLOSURE**	No, I have not used AI
**DATE REVIEW RETURNED**	12-Mar-2026

**GENERAL COMMENTS**	Thank you for letting me review this paper on an important topic. I find the study well written, interesting and well conducted. The authors have conducted a cross-sectional study, with data on adolescents from a population-based setting (via schools). They differentiate between physical illness, mental illness, substance abuse and died (RSHC) for the severely ill family member and they use a validated instrument for assessing mental well-being (MHC-SF). Additionally, they include several interesting covariates. Several statistical analyses were conducted, adjusted linear regression modelling was the most advanced. The study contributes to the understanding of the mental well-being among adolescents being in vulnerable situation in life, particularly for those having a RSHC with substance abuse.

	I only have a few comments that I hope might further improve this manuscript. Good luck with the revision!<br><br>1. It is easy to get the impression that that the adolescents are living in the same household as the relative with severe health condition. For example: in you first aim, Page 7 line 2 “to describe the prevalence of adolescents living with RSHC”. While the third aim was “to explore the association between having a RSHC<br><br>The question used to assess RSHC was “Do you have a family member or a person close to you that has… Based on this question I don’t think that you can conclude that the adolescents live together with their RSHC.<br><br>Most of the time you use “severe illness” (e.g. Page 12 line 4) for describing RSHC, but sometimes you use “serious illness” (e.g. page 15 line 50).<br><br>2. A rather large part of the introduction, page 5 lines 17-54 concerns young carers, however this is something that you do not assess in this study. Perhaps this section can be shorter in favour for the covariates that you do assess (see point6). You seem to lack references in the introduction, page 5 lines 47-48.<br><br>3. In the introduction, page 7 line 11 you state that you aim to analyse adolescent characteristics, socio-economic status. But as I understand it, you analyse the adolescent’s experience of economic stress. Perhaps it’s better to use one concept? Socio-economic status among adolescents if often based on the parents level of education or of income.<br><br>4. The study design, did all schools in the county of Örebro participate in the Life and Health Youth Survey? I get that impression from the methods section from page 7 line 34 “The survey was designed as a total population study and was distributed to all students attending the 9th…<br><br>5. Page 8 line 11 “mental well-being due to incomplete data regarding mental wellbeing” different spelling on well-being.<br><br>6. Statistical analysis, page 10 line 14. You state that you will adjust for physical disability and neurodevelopmental disability. I think that these variables needs to be mentioned earlier in the manuscript. My understanding is that age, gender, income and level of education are considered traditional sociodemographic characteristics, in this manus you include disabilities in this concept. Perhaps you could elaborate on this?<br><br>7. Did you check for multicollinearity? I’m particularly interested of the nature of the relation between RSCH and feeling stressed. In the introduction, page 6 Line 2 “Persistent worry and concern for a close relative’s health may further compromise concentration and heighten stress.” you imply that this might be the case.<br><br>8. One of your main findings is that adolescents having close relatives with substance abuse had the strongest association to lower well-being. While having a friend to confide in may be a protective factor and that adolescents having RSHC interact with

	everyday stressors. Are there any existing programs or treatments for adolescents with RSHC?

VERSION 1 – AUTHOR RESPONSE

Reviewer 1

1. is the "close relative" identified as the primary carer ofr the adolescent.

Response:

The survey item did not specify the role of the relative (e.g. primary caregiver) and may therefore include a range of close relationships. We have clarified this in the Methods section and further discussed this as a limitation of the measure in the Discussion.

2. Given the response rate, is the catchment area socio-economically disadvantaged? Is this a culturally and linguistically diverse cohort?

Response:

The study is based on a population-based survey including all eligible students in the county, with a relatively high response rate. The study population is predominantly Swedish born, with a smaller proportion of adolescents born outside Sweden, reflecting the demographic composition of the county. As described in the Discussion (Strengths and limitations), the findings should be interpreted in relation to this context. We have retained this balanced interpretation, as the study focuses on associations between variables rather than prevalence estimates, which supports generalisability of the observed patterns.

Reviewer 2

1. It is easy to get the impression that that the adolescents are living in the same household as the relative with severe health condition. For example: in you first aim, Page 7 line 2 "to describe the prevalence of adolescents living with RSHC". While the third aim was "to explore the association between having a RSHC. The question used to assess RSHC was "Do you have a family member or a person close to you that has… Based on this question I don't think that you can conclude that the adolescents live together with their RSHC. Most of the time you use "severe illness" (e.g. Page 12 line 4) for describing RSHC, but sometimes you use "serious illness" (e.g. page 15 line 50).

Response

We agree that the original wording could be interpreted as implying co-residence. We have therefore revised the manuscript to consistently refer to adolescents with RSHC rather than "living with RSHC" throughout the text. We have also clarified in the Methods section that the survey item did not specify the role or living arrangement of the relative, and may therefore include a range of close relationships rather than necessarily a co-residing family member. In addition, we have standardised the terminology throughout the manuscript to consistently use "severe health conditions" in line with the definition of RSHC.

2. A rather large part of the introduction, page 5 lines 17-54 concerns young carers, however this is something that you do not assess in this study. Perhaps this section can be shorter in favour for the covariates that you do assess (see point6). You seem to lack references in the introduction, page 5 lines 47-48.

Response

We agree that the original introduction placed too much emphasis on young carers relative to the scope of the study. We have therefore shortened this section and refocused it to more clearly emphasise adolescents with RSHC regardless of caregiving involvement. In addition, we have clarified the distinction between caregiving and the broader RSHC context to better align the introduction with the aims and analyses of the study. We have also reviewed the references where needed to support the relevant statements in this section.

3. In the introduction, page 7 line 11 you state that you aim to analyse adolescent characteristics, socio-economic status. But as I understand it, you analyse the adolescent's experience of economic stress. Perhaps it's better to use one concept? Socio-economic status among adolescents if often based on the parents level of education or of income.

Response:

We agree that the original wording could be misleading. We have therefore revised the manuscript to consistently refer to economic stress, which more accurately reflects the measure used in the study. This change has been made in the Introduction and throughout the manuscript.

4. The study design, did all schools in the county of Örebro participate in the Life and Health Youth Survey? I get that impression from the methods section from page 7 line 34 "The survey was designed as a total population study and was distributed to all students attending the 9th…

The survey was designed as a total population study and was distributed to all eligible students in the county. Eligibility was restricted to students in ordinary school programmes, meaning that students in special education schools or introduction programmes were not included. All schools were invited to participate, please see the Methods section, under Study design.

5. Page 8 line 11 "mental well-being due to incomplete data regarding mental wellbeing" different spelling on well-being.

Response

Now changed.

6. Statistical analysis, page 10 line 14. You state that you will adjust for physical disability and neurodevelopmental disability. I think that these variables needs to be mentioned earlier in the manuscript. My understanding is that age, gender, income and level of education are considered traditional sociodemographic characteristics, in this manus you include disabilities in this concept. Perhaps you could elaborate on this?

Response:

We agree that physical and neurodevelopmental disabilities are not traditionally considered sociodemographic characteristics. In this study, these variables were included as individual-level factors relevant to inequalities in adolescent mental well-being, as they are known to be associated with differences in health outcomes. We have clarified this in the Methods section (Measures and variables), where we now explicitly state the rationale for including these variables as part of the broader set of covariates.

7. Did you check for multicollinearity? I'm particularly interested of the nature of the relation between RSCH and feeling stressed. In the introduction, page 6 Line 2 "Persistent worry and

concern for a close relative's health may further compromise concentration and heighten stress." you imply that this might be the case.

Response:

Multicollinearity was assessed using variance inflation factors (VIF), and no evidence of problematic multicollinearity was observed (all VIF values <1.3). This has now been clarified in the Methods section, under Statistical analysis. Regarding the relationship between RSHC and stress, we agree that this is conceptually relevant. However, in the present study, stress was included as a separate covariate to capture the broader psychosocial context. The low VIF values indicate that RSHC and stress were not highly collinear, suggesting that their associations with mental well-being can be interpreted as independent contributions within the model.

8. One of your main findings is that adolescents having close relatives with substance abuse had the strongest association to lower well-being. While having a friend to confide in may be a protective factor and that adolescents having RSHC interact with everyday stressors. Are there any existing programs or treatments for adolescents with RSHC?

Response:

We agree that our findings have clear implications for intervention and support. We have therefore expanded the Discussion to situate our results in relation to existing intervention research. We note that existing interventions have primarily focused on specific subgroups, such as young carers or children of parents with mental illness or substance use problems, including psychosocial, family-based and school-linked support programmes. However, fewer interventions appear to target adolescents with RSHC as a broader and more heterogeneous group. This addition has been incorporated into the Discussion section to highlight both existing support approaches and the need for further development of strategies that reflect the diversity of adolescents' experiences and the importance of psychosocial context and emotional support.

VERSION 2 – REVIEW

**REVIEWER NAME**	Thazhissery, Anjana
**REVIEWER AFFILIATION**	Sydney South West Area Health Service, ICAMHS
**REVIEWER CONFLICT OF INTEREST**	No Conflict of Interest
**AI DISCLOSURE**	No, I have not used AI
**DATE REVIEW RETURNED**	27-Apr-2026

**GENERAL COMMENTS**	Thank you for addressing the points raised.

**REVIEWER NAME**	Tiikkaja, Sanna
**REVIEWER AFFILIATION**	Uppsala University Department of Public Health and Caring Sciences, CHAP
**REVIEWER CONFLICT OF INTEREST**	No Conflict of Interest
**AI DISCLOSURE**	No, I have not used AI
**DATE REVIEW RETURNED**	22-Apr-2026

**GENERAL COMMENTS**	The authors have responded satisfactorily to my comments, and I think that revisions have strengthened and improved the manuscript. I have no further comments.
